# Whole Genome Level Analysis of the Wnt and DIX Gene Families in Mice and Their Coordination Relationship in Regulating Cardiac Hypertrophy

**DOI:** 10.3389/fgene.2021.608936

**Published:** 2021-06-08

**Authors:** Zhongchao Gai, Yujiao Wang, Lu Tian, Guoli Gong, Jieqiong Zhao

**Affiliations:** ^1^School of Food and Biological Engineering, Shaanxi University of Science and Technology, Xi’an, China; ^2^Department of Cardiology, The Second Affiliated Hospital of Air Force Medical University, Xi’an, China

**Keywords:** gene family, evolution, gene expansion, cardiac hypertrophy, heart development

## Abstract

The Wnt signaling pathway is an evolutionarily conserved signaling pathway that plays essential roles in embryonic development, organogenesis, and many other biological activities. Both Wnt proteins and DIX proteins are important components of Wnt signaling. Systematic studies of Wnt and DIX families at the genome-wide level may provide a comprehensive landscape to elucidate their functions and demonstrate their relationships, but they are currently lacking. In this report, we describe the correlations between mouse Wnt and DIX genes in family expansion, molecular evolution, and expression levels in cardiac hypertrophy at the genome-wide scale. We observed that both the Wnt and DIX families underwent more expansion than the overall average in the evolutionarily early stage. In addition, mirrortree analyses suggested that Wnt and DIX were co-evolved protein families. Collectively, these results would help to elucidate the evolutionary characters of Wnt and DIX families and demonstrate their correlations in mediating cardiac hypertrophy.

## Introduction

The Wnt signaling pathway is a highly conserved signaling pathway that plays crucial roles in various life activities in vertebrates and invertebrates, such as embryonic development, organogenesis, neurological development and inflammation (Clevers and Nusse, 2012). The Wnt pathway can be divided into three branches based on the signaling transduction cascade: the β-catenin pathway, the planar cell polarity (PCP) pathway, and the Wnt/Ca^2+^ pathway. The first branch is known as the canonical Wnt pathway, and the last two branches are classified into the non-canonical Wnt pathways. Both of these two non-canonical pathways inhibit canonical signaling (Daulat and Borg, 2017; Vargas et al., 2019; Jackson et al., 2020). Canonical Wnt signaling regulates the pluripotency of stem cells of various lineages and determines the cell fate of differentiation during development (Tao et al., 2019). It functions by regulating the transcriptional coactivator β-catenin to control key downstream gene expression programs (Macdonald et al., 2009). Wnt/PCP signaling involved in organ formation and mediates migratory events; its components are frequently dysregulated in many solid tumors (Dale et al., 2009; Vandervorst et al., 2019). Aberrant activation of Wnt/PCP signaling usually contributes to cancer cell migratory properties (Asad et al., 2014). The Wnt/Ca^2+^ pathway contributes to an increase in intracellular calcium and activation of calmodulin dependent protein kinase II (CaMKII) (Kuhl et al., 2000; Gentzel et al., 2015; Ferrari et al., 2018). Wnt/Ca^2+^ signaling modulates cell movement and induced (Witze et al., 2008; Vargas et al., 2019).

The Wnt protein is a family of secreted glycoproteins that regulate various intracellular processes (Macdonald et al., 2009). Wnt proteins are approximately 40 kDa in size and contain a Wnt domain. A mature Wnt protein contains a monounsaturated fatty acid modified serine that is necessary for the secretion of Wnt proteins (Takada et al., 2006). Wnt proteins exert crucial functions in regulating downstream signaling; mutations or aberrant expression of Wnt leads to various diseases in humans. Homozygous *Wnt1* mutation leads to osteogenesis imperfecta with exotropia and congenital ptosis in humans (Lu et al., 2018; Han et al., 2020). In odonto-onychodermal dysplasia (OODD) patents, anodontia of permanent teeth is caused by the biallelic *Wnt10A* gene mutations (Bergendal et al., 2016; Yu et al., 2019). Mutations in *Wnt9b* are frequently associated with Mayer-Rokitansky-Küster-Hauser syndrome (MRKHS) (Waschk et al., 2016). In addition, a homozygous nonsense mutation (Q83X) in the *Wnt3* gene is closely related to tetra-amelia syndrome (Niemann et al., 2004).

Axin and Disheveled act as pivotal regulators in Wnt signaling (Song et al., 2014; Sharma et al., 2018). The commonality between these proteins is that both contain the DIX domain, which consists of approximately 70 amino acids. The DIX domain is involved in homo and heterooligomerization of DIX domain-containing proteins. The oligomerization of disheveled/Axin affects the transduction of Wnt/β-catenin signaling (Kan et al., 2020). Frame shift mutation decreases its ability to undergo Wnt-induced phosphorylation and further alters Wnt signaling, eventually leading to Robinow-like syndrome in bulldogs (Mansour et al., 2018). Mutations in AXIN1/2 genes have been proven to be related to hepatocellular carcinoma, prostate cancer and lung cancer (Taniguchi et al., 2002; Koppert et al., 2004; Tseng et al., 2008; Kim et al., 2009). The downregulation of Axin correlates with the cancer progression of esophageal squamous cell carcinoma (OSCC) (Nakajima et al., 2003). Moreover, the methylation status of Axin genes correlates with the radiosensitivity of lung cancer cells (Yang et al., 2013).

Many studies suggest that Wnt signaling is closely related to the embryonic heart development and some cardiac diseases of vertebrates, and Wnt proteins have also been proven to play various important roles during cardiac differentiation and development (Gessert and Kuhl, 2010). Wnt/β-catenin signaling enhances stem cell expression in the developing heart of the frogs, without inhibiting of cardiogenic differentiation (Martin et al., 2011). The β-catenin protein controls ventricular myocyte proliferation, and Wnt/β-catenin signaling accounts for the different proliferation rates of the compact versus trabecular myocardium during embryonic heart development (Buikema et al., 2013). In vertebrates, Wnt signaling not only controls heart development but also modulates adult heart remodeling (Mazzotta et al., 2016; Jeong et al., 2017; Bornhorst et al., 2019). miR-29 promotes pathological remodeling of the heart by activating Wnt signaling in mice (Sassi et al., 2017).

The genome-wide analysis of a specific gene family can provide us with a systematic understanding of the characteristics of its family members, including classification, gene expression and molecular evolution (Gai and Zhao, 2020; Zhao et al., 2020b). However, relating studies are currently lacking for Wnt and DIX gene families in mammal lineage. Moreover, both Wnt and DIX genes are crucial players in Wnt signaling, but the relationships between them in genetic evolution and regulation of cardiac hypertrophy remain obscure. In consideration of their important roles in various life activities and their abundance, it is necessary to perform a genome-wide analysis of Wnts and DIXs. In the present work, we identified Wnts and DIXs separately in mice (*Mus musculus*) and other species at the genome-wide level. Then the chromosomal distributions, phylogenetic and evolutionary relationships of mouse Wnt and DIX family members were identified. Furthermore, we analyzed the expression profiles of Wnt and DIX genes in the heart of cardiac hypertrophic mice. Finally, we studied the 3-dimensional (3D) structures of Wnt and DIX proteins and explored the conservation of Wnt and DIX domains from different species separately. This work provided an in-depth understanding of the Wnt and DIX protein families in the mammalian lineage and demonstrated their correlation in mediating the progression of cardiac hypertrophy.

## Results

### Identification and Characterization of Wnt and DIX Family Members

The hidden Markov model (HMM) profiles of the Wnt domain (PF00110) and DIX domain (PF00778) were used to identify all Wnt or DIX domain-containing proteins in the mouse (*M. musculus*) genome; all identified candidates were further confirmed in the InterPro database. A total of 19 non-redundant Wnt family members and 6 DIX family members were identified in the mouse genome. Detailed information of Wnt and DIX family members is listed in Supplementary Tables 1, 2. The mRNA lengths of these 19 Wnt genes ranged from 3,974 bp (Wnt1) to 111,817 bp (Wnt5b) with ORFs ranging from 1,050 bp (Wnt7a) to 1,254 bp (Wnt10a). The Wnt proteins ranged from 417 aa (Wnt10a) to 349 aa (Wnt7a) in length. The isoelectric points (pIs) of all Wnt proteins were alkaline, varying from 9.26 (Wnt10b) to 7.51 (Wnt3). On the other hand, the mRNA lengths of these six DIX genes ranged from 57,123 bp (Axin1) to 9,517 bp (Dvl2) with ORFs ranging from 2523 bp (Axin2) to 2088 bp (Dvl1). The DIX proteins ranged from 840 aa (Axin2) to 695 aa (Dvl1) in length. The isoelectric points (pIs) of all DIX proteins were close to neutral, varying from 7.80 (Axin2) to 5.82 (Dvl2).

**FIGURE 1:**
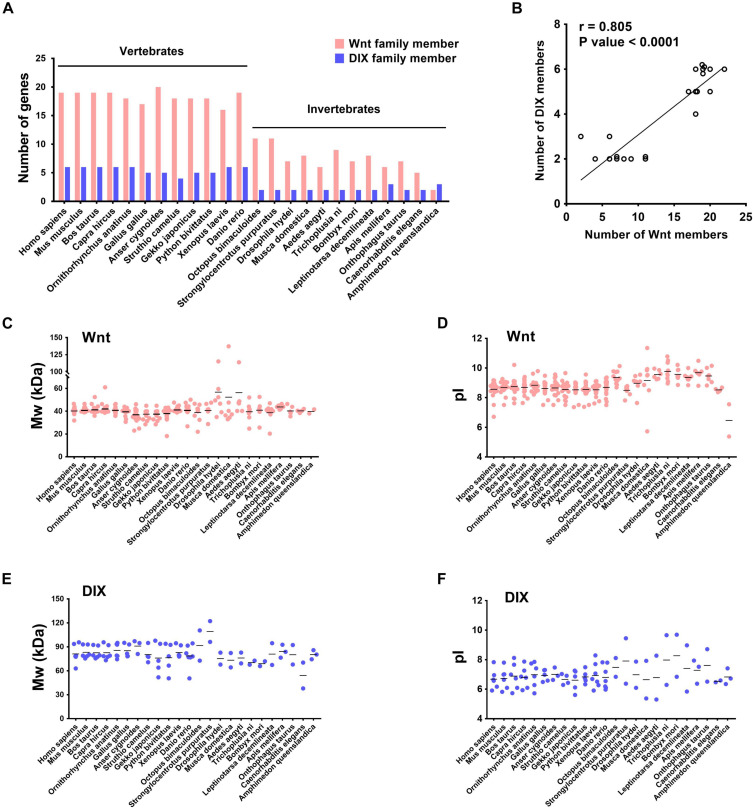
The quantities and physicochemical characteristics of Wnt and DIX family members in different species. **(A)** The Wnt and DIX genes in 12 vertebrates and 12 invertebrates were identified. The number of Wnt genes in vertebrates (mean ∼18) is larger than that in invertebrates (mean ∼7). Similarly, the average number of DIX genes in vertebrates was 6, and in invertebrate, it was 2. **(B)** Pearson correlation of the number of the Wnt and DIX genes in the process of species evolution. Molecular weight distributions of Wnt proteins **(C)** and DIX protein **(E)** from different species. Isoelectric point (pI) distributions of Wnt proteins **(D)** and DIX proteins **(F)** from different species.

After identifying Wnt and DIX genes in the mouse, we further identified them in 23 other species, including 11 vertebrates and 12 invertebrates. In general, there were more Wnt and DIX genes in vertebrates, and their quantities increased with the evolutionary process (Figure 1A). The results of the Pearson correlation test indicated that the gene expansion of Wnt and DIX genes was highly correlated (*r* = 0.805, *P* < 0.0001) (Figure 1B). Furthermore, we analyzed the molecular weights and pI values of all 312 Wnt proteins and 92 DIX proteins from 24 species. The results showed that the molecular weights of Wnt proteins in *Drosophila hydei*, *Musca domestica*, and *Aedes aegypti*, all of them were dipterans, were significantly larger than those of the other 21 species (Figure 1C and Supplementary Figure 1A). This was because their genome contains a larger Wnt-5 coding gene. The theoretical pI values of Wnt proteins from most invertebrates here studied were significantly higher than those of 12 vertebrates studied here (Figure 1D and Supplementary Figure 1B). However, there was no significant difference between the theoretical pI values and molecular weights of DIX proteins from all 24 species studied here (Figures 1E,F and Supplementary Figures 1C,D).

### Local Accumulation of Mouse Wnt Genes on Certain Chromosomes

**FIGURE 2:**
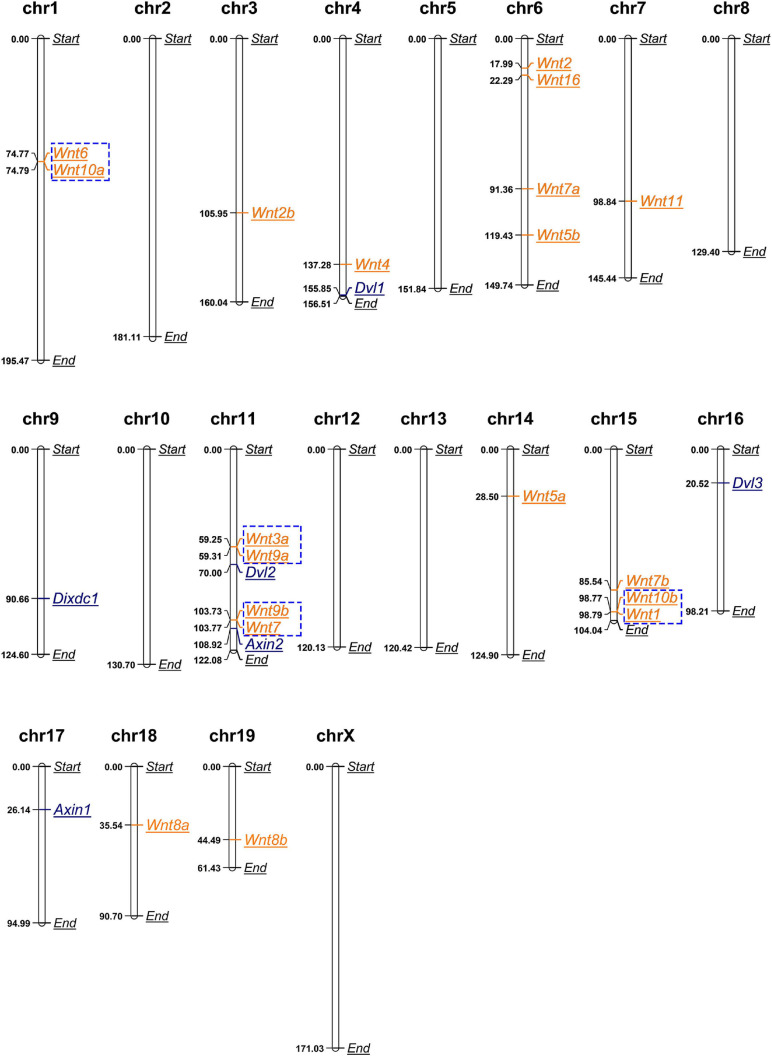
Mouse Wnt genes locally accumulate in certain chromosomes. Chromosomal distributions of mouse Wnt and DIX genes. Genes in blue boxes are tandem duplication genes. The length unit for all chromosomes shown here is megabase (Mb).

We obtained the chromosomal coordinates of mouse Wnt and DIX genes from the MGI database and then both Wnt and DIX genes were mapped to the chromosomes of the mouse genome (Figure 2). This coordinate map showed that out of the 19 Wnt genes, 11 (58%) are locally accumulated on the three chromosomes (chromosome 6, 11, and 16). Chromosomes 6 and 11 had four (21%) Wnt genes, chromosome 15 had 3 (16%) Wnt genes. Chromosome 3, 4, 7, 18, and 19 each contain one Wnt gene. Chromosome 1 contains two Wnt genes. All six DIX genes were distributed in five chromosomes, and Dvl2 and Axin2 were located on chr11, which also contained the most Wnt genes. Furthermore, we determined the gene duplication of Wnt genes on the mouse chromosomes. The results showed that eight Wnt genes were tandem repeat genes, which accounted for 42% of the total Wnt genes. These genes can be divided into four groups, including group I (Wnt6 and Wnt10a) located on chromosome 1, group II (Wnt3a and Wnt9a) and group III (Wnt9b and Wnt7) located on chromosome 11, and group IV (Wnt10b and Wnt11) located on chromosome 15, implying that the tandem repeats events contributed to the gene expansion of Wnt genes in mouse. However, it was notable that there were no tandem repeat genes in the DIX family.

### Phylogenetic Analyses of Wnt and DIX Family Members

**FIGURE 3:**
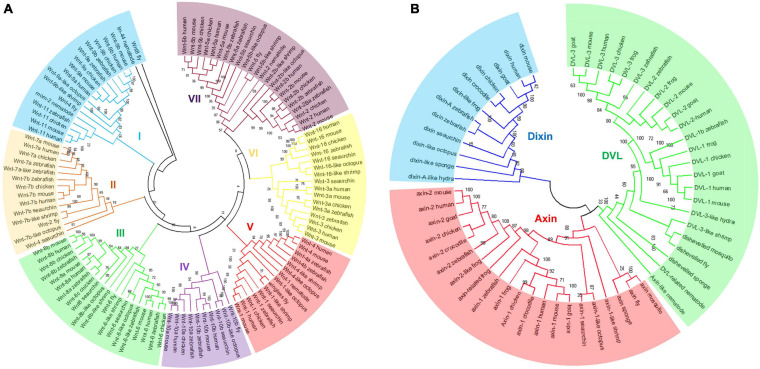
Phylogenetic classification of Wnt and DIX proteins. The phylogenetic trees of Wnt **(A)** and DIX **(B)** were built through the neighbor-joining (N-J) method with 1000 bootstrap replicates. All Wnt proteins from different species were divided into seven groups, and the DIX proteins were divided into three major groups. Both of these major groups are highlighted in different colors.

To investigate the phylogenetic relationships of Wnt and DIX genes between mouse and other species, the amino acid sequences of 118 Wnt proteins and 57 DIX proteins from mouse and the other species were separately aligned using the ClustalW method, and then neighbor-Joining (N-J) phylogenetic trees were constructed based on sequence alignments. The results showed that Wnt proteins could be divided into seven major groups in evolution. The cluster VII was the most abundant, including 23 Wnt genes from human (4), mouse (4), zebrafish (4), chicken (4), octopus (2), sea urchin (1), shrimp (1), fly (1) and nematode (1), accounting for nearly 19.5% of all the Wnt genes here studied. Cluster III accounted for 16.9%, with 20 Wnt genes. Cluster I accounted for 15.3%, with 18 Wnt genes. Cluster IV had the smallest number, with only 10 Wnt genes, accounted for 8.5% (Figure 3A). All 57 DIX genes here studied were divided into three groups: Dixin, Axin and Dvl. The Dvl group was the most abundant, accounted for 42.1%, with 24 DIX genes. The Axin group contained 21 members, accounted for 36.8%. The Dixin group was the smallest group, contained 12 members, accounted for 21.1% (Figure 3B).

### Both the Wnt and DIX Families Underwent More Expansion Than the Overall Average in the Evolutionarily Early Stage

Studying the expansion of the Wnt and DIX families in the context of an evolutionary tree with pivotal time points can help disclose their evolutionarily characteristics, as different members of the mouse Wnt and DIX protein families should have emerged at different evolutionary stages, and may exert different biological functions.

**FIGURE 4:**
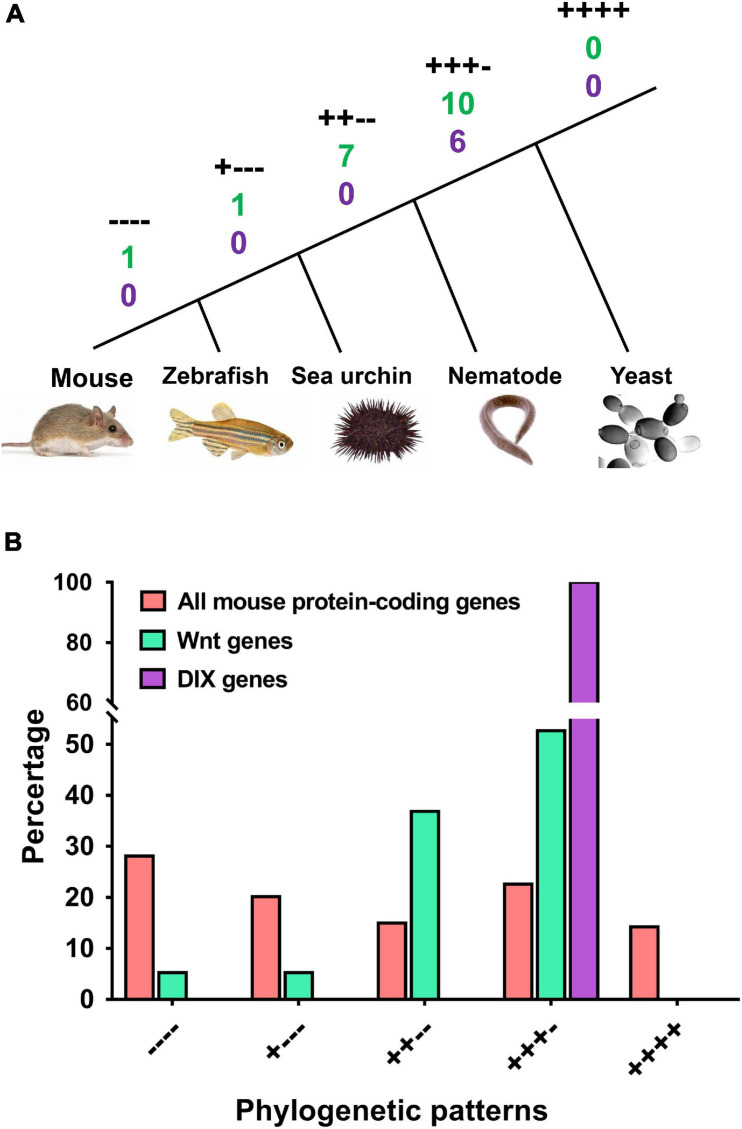
Both the Wnt and DIX families underwent more expansion than the overall average in the evolutionarily early stage. The phylogenetic patterns of mouse Wnt and DIX genes and all mouse protein-coding genes were compared. **(A)** Phylogenetic relationships of five representative species, including mouse, zebrafish, sea urchin, nematode and yeast. The symbols “++++,” “+++ –,” “++ – –,” “+ – – –,” and “– – – –” denote the different phylogenetic patterns. The numbers under each label denote the quantities of Wnt (green) and DIX (purple) genes accordingly. **(B)** Comparison between the proportions of mouse Wnt and DIX genes and all mouse protein-coding genes in different phylogenetic patterns.

Here, we performed a phylogenetic analysis according to the Wnt orthologs in four model organisms, including yeast, nematodes, sea urchins and zebrafish. These organisms are representative of single-celled eukaryotes, lower multicellular organisms, invertebrates, and vertebrates. The appearance of these four organisms represents several key time points in the evolutionary tree (Figure 4A). The mouse Wnt and DIX proteins with orthologs in all other four species were labeled ‘++++,’ which indicated that they emerged with the origin of eukaryotes. However, we found that no Wnt or DIX orthologs were identified in yeast (*Saccharomyces cerevisiae*). Ten Wnt (52.6%) and six DIX (100%) proteins in mice have orthologs in nematodes (*Caenorhabditis elegans*) and labeled ‘+++ −,’ and this result suggests that more than half of the members of the Wnt and DIX families emerged with the appearance of multicellular organisms. Seven mouse Wnt proteins (36.8%) had orthologs only in sea urchins (labeled ‘++ −−’), indicating that they may have emerged before the separation of invertebrates and vertebrates. One Wnt protein has an ortholog only in zebrafish (*Danio rerio*), suggesting that it may emerge before the separation of aquatic vertebrates and terrestrial vertebrates. Additionally, there is one Wnt family member without ortholog in any of the other four species, which illustrates that it should have originated with the lineage expansion of terrestrial vertebrates (labeled ‘− − − −’).

When comparing mouse Wnt and DIX family genes with all mouse protein-coding genes, we found that a larger proportion of Wnt and DIX genes than that of all genes (52.63% vs. 36.80% for Wnt and 100% vs. 36.80% for DIX) should have originated at the very early stage of eukaryotes (Figure 4B and Supplementary Table 3). Only 10.52% of mouse Wnt genes emerged after the separation of invertebrates and vertebrates, however, nearly half of the total protein coding genes (48.22%) in mice emerged in this period.

### Domain Organization Analysis of Mouse Wnt and DIX Family Members

**FIGURE 5:**
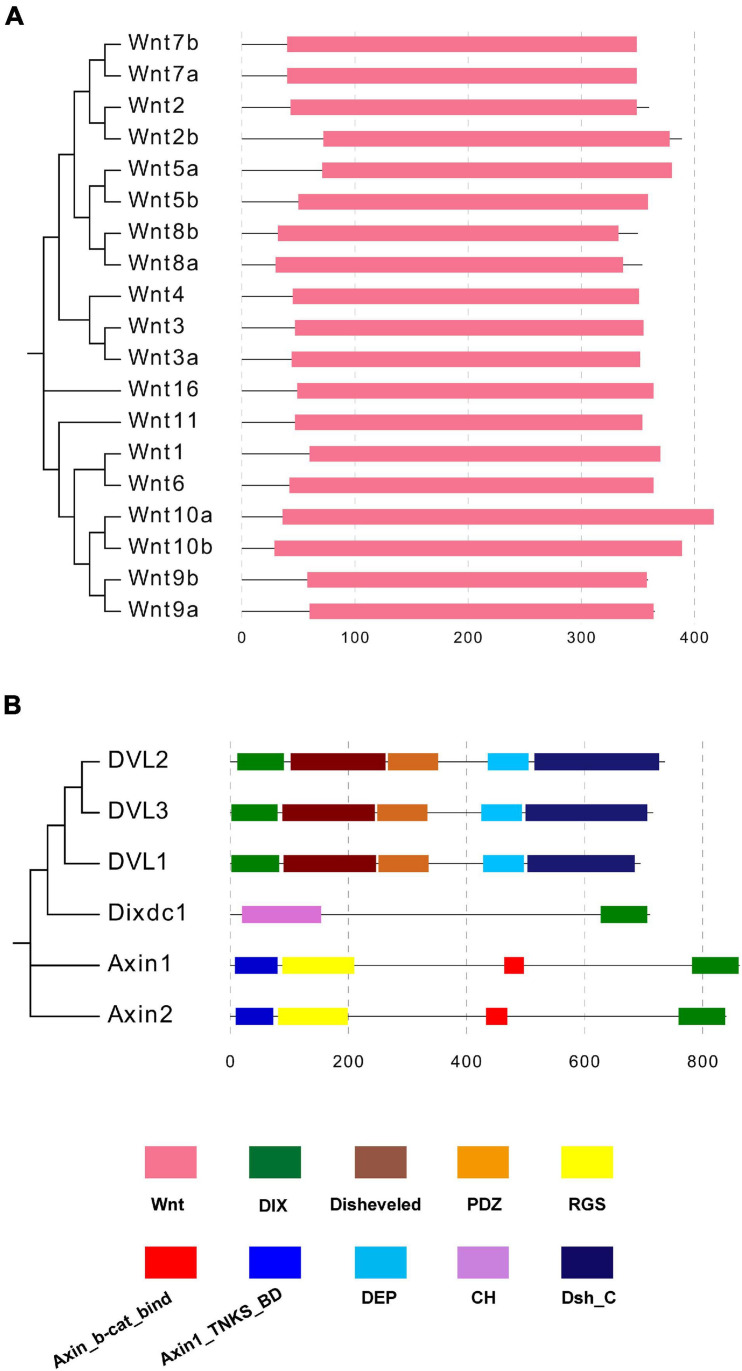
**(A,B)** Domain compositions of mouse Wnt and DIX proteins. Domain organization information of mouse Wnt and DIX proteins was obtained in the Pfam database and domains are marked in different colors.

A phylogenetic tree was constructed based on the 19 mouse Wnt proteins via the N-J method with 1000 bootstraps (Figure 5A). Then the domain organization information obtained from the Pfam database showed that the Wnt domain was the unique functional component of all 19 mouse Wnt proteins. Compared with Wnt proteins, mouse DIX proteins contain various functional domains, including DIX, Dvl, PDZ, RGS, and DEP (Figure 5B). It was notable that the DIX domains located at the N terminal of Dvl1/2/3, in contrast, they were located at the C terminal of Dixin and Axin1/2. These functional domains contributed to the functional diversity of DIX proteins. Moreover, the domain organizations of Dvl1/2/3 were more compact than those of Axin1/2 and Dixdc1 (Dixin).

### Wnt and DIX Were Co-evolved Protein Families

**FIGURE 6:**
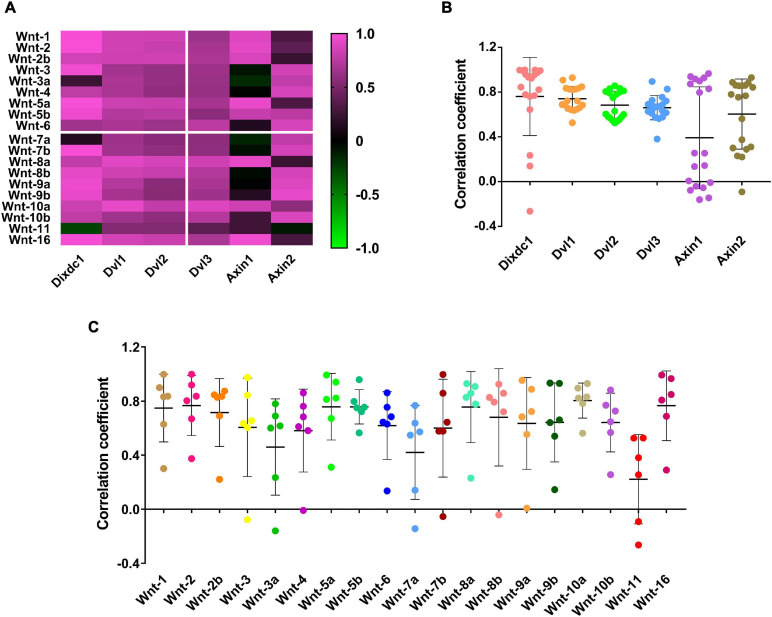
Wnt and DIX were co-evolved protein families. **(A)** The co-evolution correlation matrix between 19 Wnt proteins and 6 DIX proteins. **(B)** The scatter plots exhibit the Wnt-DIX co-evolution correlation of six DIX members. **(C)** The scatter plots exhibit the Wnt-DIX co-evolution correlation of 19 Wnt members.

The Mirrortree server was used to investigate the co-evolution relationship between the Wnt and DIX family members at the molecular level. The Pearson correlation of co-evolution of 19 Wnt proteins and 6 DIX proteins were calculated by the MirrorTree are shown in Supplementary Table 4. These results showed that the co-evolution between most Wnt and DIX proteins were positive correlations, and the mean value of Pearson correlation is 0.641 (Figure 6A). It was noticeable that the Pearson correlation coefficients between DVL1/2/3 and Wnt proteins were very similar, ranged from 0.381 (DVL3-Wnt11) to 0.930 (DVL1-Wnt10a). Compare to other DIX proteins, the Pearson correlation coefficients between Axin1 and Wnt proteins were the lowest (Figure 6B). Moreover, the Pearson correlation coefficients between Wnt11 and DIX proteins were lower than other 18 Wnt proteins (Figure 6C). Overall, the co-evolution analyses suggested that Wnt proteins were evolutionarily associated with DIX proteins.

### Wnt and DIX Genes Participate in Mediating Cardiac Hypertrophy in Similar Expression Patterns

**FIGURE 7:**
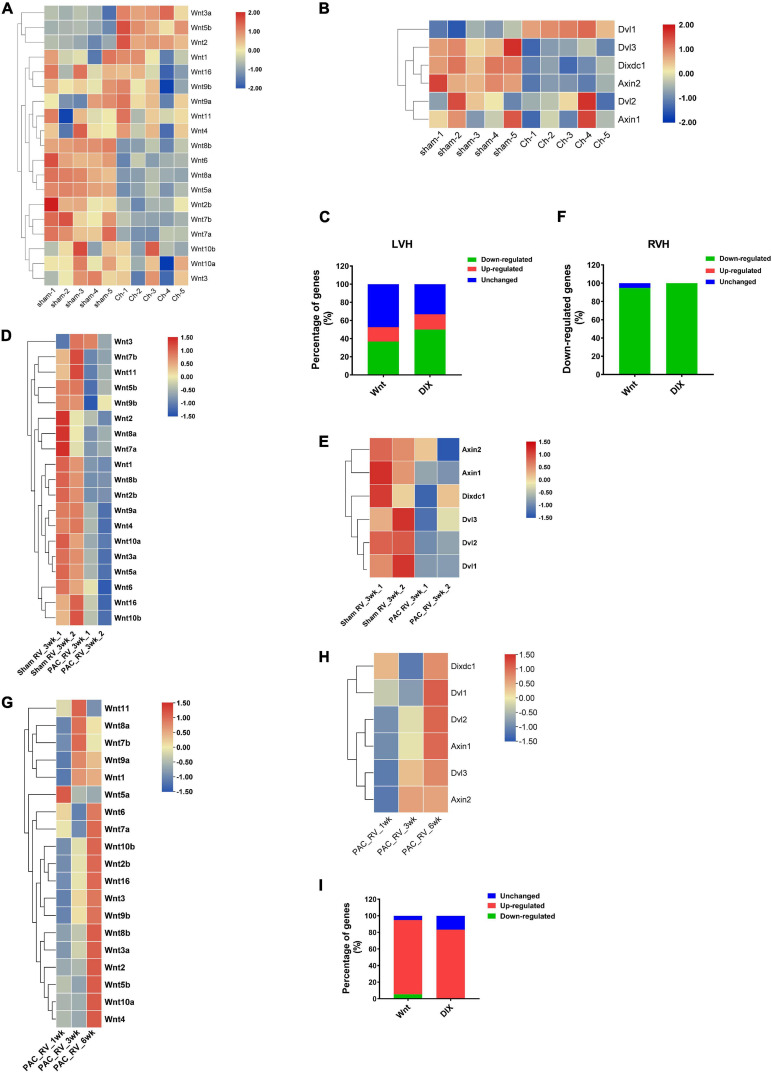
The expression patterns of mouse Wnt and DIX genes in left and right ventricle hypertrophy. Expression patterns of Wnt genes **(A)** and DIX genes **(B)** in the left ventricle of the mouse heart. Sham indicates sham operations, Ch indicates TAC operations induced chronic hypertrophy. The color scale bars represent the differential expression levels of each gene. **(C)** The ratio of downregulated Wnt and DIX genes in the left ventricle of mice after TAC or Sham operations. **(D)** Expression patterns of Wnt genes and DIX genes **(E)** in the right ventricle of the mice heart. Sham indicates sham operations, PAC indicates pulmonary artery clipping operations. 3wk means 3 weeks after operations. **(F)** The expression change of Wnt and DIX genes in the right ventricle of mice when 3 weeks after PAC operations. **(G)** Expression patterns of Wnt genes and DIX genes **(H)** in the right ventricle of the mice heart when 1 week, 3 weeks and 6 weeks after PAC operations. **(I)** The expression change of Wnt and DIX genes in the right ventricle of mice when 1, 3, and 6 weeks after PAC operations. All heatmaps were generated by using the pheatmap package in R software (version 4.0). The color scale bars represent the differential expression levels of each gene.

Previous studies have suggested that many Wnt family members are involved in mediating the progression of left ventricle hypertrophy (LVH). Here, we analyzed the transcriptomic data of the left ventricles of transverse aortic constriction (TAC) induced cardiac hypertrophic mice, and the results showed that many Wnt (52.63%) and DIX (66.67%) genes exhibited differential expression patterns when 4 weeks after TAC operations. In Wnt family, seven (36.84%) members were down-regulated, three (15.79%) members were upregulated and the others (47.37%) were not changed (Figure 7A). In DIX family, three (50%) members were downregulated after TAC, one (16.67%) member was upregulated and the other two members were not changed (Figure 7B). Compared to LVH, there is less information about the pathogenesis mechanism of right ventricle hypertrophy (RVH). We analyzed a set of gene expression data of the right ventricle of cardiac hypertrophic mice model, which was generated through pulmonary artery clipping (PAC) methods. The results showed that nearly all members of mouse Wnt family, except for Wnt3, were downregulated in the right ventricle of RVH mice when 3 weeks after PAC operation (Figure 7D). Interestingly, all DIX family members were also downregulated in the right ventricle of RVH mice (Figure 7E). The statistical results suggested that both Wnt and DIX genes exhibited similar expressional patterns in TAC or PAC induced cardiac hypertrophy (Figures 7C,F). In addition, we analyzed the expression patterns of Wnt genes and DIX genes in the right ventricle of the mice heart when 1 week, 3 weeks and 6 weeks after PAC operations. Results showed that most Wnt genes and DIX genes were upregulated as time went on (Figures 7G,H,I).

### The 3D Structure of Both Wnt and DIX Domains Are Highly Conserved in Evolution

**FIGURE 8:**
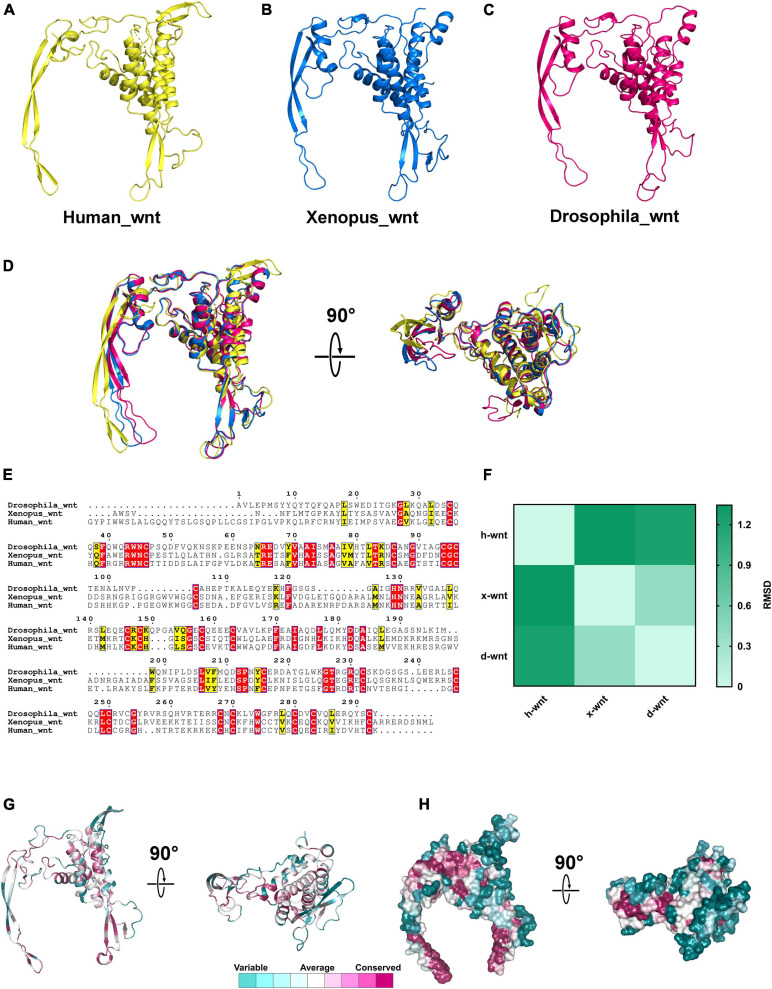
The 3D structures of Wnt proteins in different species. **(A–C)** 3D structures of Wnt domains from humans, *Xenopus* and *Drosophila*. **(D)** Structural alignments of Wnt domains from different species; the colors correspond to panels **(A,B,C)**. **(E)** Amino acids sequences alignment of Wnt domains. **(F)** RMSD matrix of alignments of Wnt domains from different organisms. **(G,H)** Residue and surface conservation of the mouse Wnt domain.

Various functional protein domains that play important roles in biological processes are evolutionarily conserved in both primary and tertiary structures, such as the ACT domain, WD40 domain, and PDZ domain (Zhao et al., 2020b). To investigate the structural characteristics of Wnt and DIX domains between different species, we downloaded the 3D structures of Wnt domains of humans (PDB id: 6ahy), Xenopus (PDB id: 4f0a) and Drosophila (PDB id: 4krr) from the PDB database (Janda et al., 2012; Chu et al., 2013; Hirai et al., 2019). The 3D structures of Wnt domains showed that they were both composed of approximately 6–8 α-helixes and 4–6 β-strands, and all components were arranged in similar patterns (Figures 8A–C). The 3D structural alignments showed that Wnt domains from three different species were similar in spatial conformation, and the root-mean-square deviations (RMSD) ranged from 0.387 to 1.345, which suggested that Wnt domains were structurally conserved during evolution (Figures 8D,F). Notably, the amino acid sequences of Wnt domains from humans, Xenopus and Drosophila were not as highly conservative as 3D structures, and identical residues accounted for only 13.3% of all aligned residues (Figures 8E,G,H).

**FIGURE 9:**
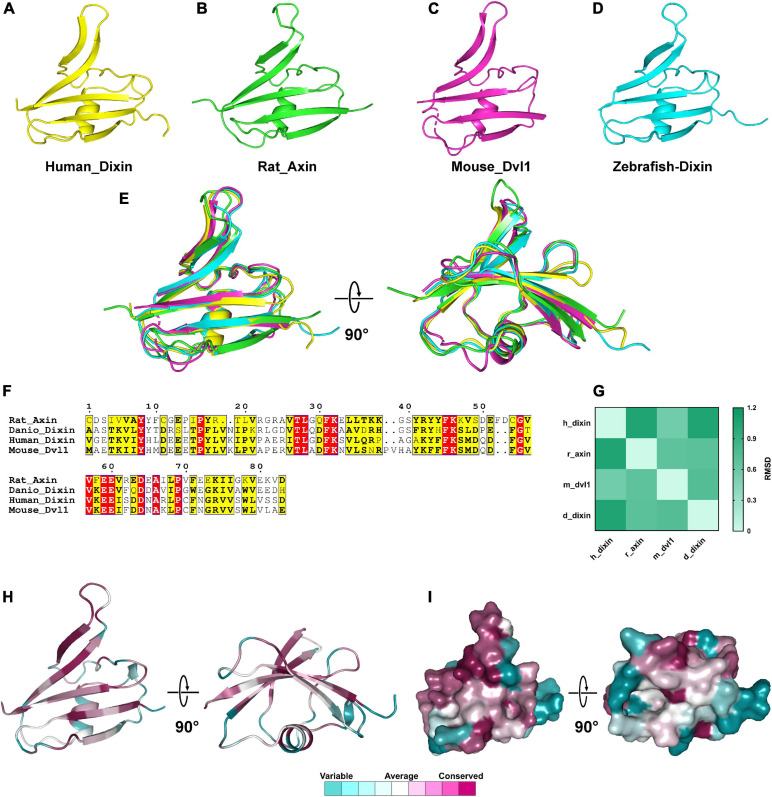
The 3D structures of DIX domains from different species are evolutionarily conserved. **(A–D)** 3D structures of Wnt domains from human, rat, mouse and zebrafish. **(E)** Structural alignments of Wnt domains from different species and the colors correspond to panels **(A,B,C,D)**. **(F)** Amino acids sequences alignment of DIX domains. **(G)** RMSD matrix of alignments of DIX domains from different species. **(H,I)** Residue and surface conservation of the mouse DIX domain.

Compared to the Wnt domain, the DIX domains contain fewer residues. The 3D structures of the DIX domains of rat Axin_DIX (1wsp), zebrafish Dixin_DIX (5y3c), mouse Dvl1_DIX (3pz8) and human Dixin_DIX (4wip) were obtained from the PDB database (Shibata et al., 2007; Liu et al., 2011; Madrzak et al., 2015; Terawaki et al., 2017). Structures showed that all these DIX domains were composed of five β-strands and one α-helix (Figures 9A–D). Further structural alignments indicated that these DIX domains were quite similar and the RMSDs between each structure ranged from 0.557 to 1.055 (Figures 9E,G). These results indicated that DIX domains were highly conserved during evolution. Moreover, the amino acid sequence alignments of DIX proteins showed that the DIX proteins were highly conserved at the primary structural level (Figure 9F). Through sequence alignments and structural analyses, we found that major conserved residues of the DIX domain were located at the β-strand region, which was mainly responsible for the protein-protein interaction (Figures 9H,I).

## Discussion

Wnt signaling pathways play crucial roles in various biological activities in animals. They mainly function in embryonic development, including organ development, cell fate decisions, cell migration and cell proliferation. Wnt- and DIX-family members are core components of Wnt signaling, and both are involved in the regulation of this important signaling pathway. However, genome-wide analysis of Wnt- and DIX-family members in mammals is still lacking. In addition, the relationship between Wnt- and DIX-family members at the genome-wide level has not been reported before. Here, we found that there are 19 Wnt genes and 6 DIX genes in the *M. musculus* genome, similar to those in other vertebrates. The total numbers of Wnt and DIX genes in mice and 11 other vertebrates are similar, which indicates that neither Wnt nor DIX gene families undergo significant expansion in the evolution of vertebrates. In addition, the total number of the Wnt and DIX genes is significantly correlated during species evolution, which also implies that there may be some potential functional coordination between Wnt and DIX genes in life activities. There was a significant difference between the pI values of vertebrates and invertebrates. A reasonable explanation is that Wnt proteins are secreted proteins, and the physicochemical properties of the extracellular matrix of invertebrates is differ from those vertebrates. To adjust the extracellular environment, the process of species evolution is also accompanied by the molecular evolution of Wnt proteins.

Various types of duplication sequences are abundant in the eukaryotic genome, and they are associated with biological processes at distinct levels. Tandem duplications and segmental duplications are the main forms of duplication genes in mammals. Other than some highly dynamic gene families, Wnt genes tended to appear in clusters in chromosomes. Our results suggest that there are 8 (4 pairs) tandemly duplicated Wnt genes, nearly half of the total. Similar duplication events have also been found in zebrafish and sea anemones (Ramel et al., 2004; Cho et al., 2010). In addition to this tandemly duplicated gene pairs, some tandem duplication gene clusters have also been found in some metazoans, such as sea anemones (Kusserow et al., 2005). All these findings indicate the high occurrence of tandem duplication events in the evolution of Wnt family members. Compared to the Wnt family, the DIX family has few members ranging from lower sponges to higher mammals, which suggests that the expansion of the DIX family was lower than that of the Wnt family.

Gene gain or loss in gene family dynamics plays an important role in evolutionarily processes (Zhang et al., 2020). Studies showed that the genomes of terrestrial mammals had largely expanded in the late stage of evolution, such as cytochrome P450 (CYP450), glutathione *S* transferase (GST) and UDP - glucuronosyltransferase (UGT) (International Human Genome Sequencing, 2004; Zhang et al., 2020). Distinct from these gene families, our results indicated that both Wnt and DIX families have undergone more expansion events than the average of all protein genes during the early evolutionarily stages. The Wnt system is an evolutionarily conserved signaling pathway that plays essential roles in cell organization and organ development during embryogenesis. In some aberrant conditions, such as organ injury and regeneration, Wnt signaling is reactivated. Earlier expansion in evolution and high evolutionary stability of Wnt and DIX genes guarantees their functional conservation in organisms. In addition, the co-evolution analyses of Wnt and DIX family members furtherly suggested their close relations in molecular evolution.

Previous reports have proven that some Wnt proteins and DIX proteins are involved in the regulation of embryonic heart development and certain cardiac diseases in adults in mammals (Kuhl et al., 2000; Martin et al., 2011; Jeong et al., 2017). Cardiac hypertrophy is a common cardiovascular disease, and chronic pathological cardiac hypertrophy will eventually lead to the heart failure (Zhao et al., 2020a). LVH and RVH are the main types of hypertrophy, more than half of the Wnt and DIX genes were differentially expressed in TAC induced LVH, and most of these differentially expressed genes were downregulated. In addition, we found that both the Wnt and DIX family members were downregulated in PAC-induced RVH. Most Wnt and DIX genes showed similar expression patterns after PAC or TAC surgical operation. In Wnt signaling pathways, Wnt proteins interact with transmembrane proteins Frizzled and active the downstream intracellular signalings. DIX proteins are important components of intracellular Wnt signaling, the expressional coordination of the Wnt and the DIX proteins contributes to the progression of cardiac hypertrophy.

## Materials and Methods

### Genome-Wide Identification of Wnt and DIX Family Members in *M. musculus*

We downloaded the whole genome sequence and all protein sequences of mouse (*M. musculus*, assembly GRCm39) from the NCBI genome database. The HMM profile of the Wnt domain (PF00110) and DIX domain (PF00778) were downloaded from the Pfam database^1^ (El-Gebali et al., 2019). The hmmsearch program (E-value ≤ 1 × 10^–5^, score ≥ 0) in the HMMER package (version 3.2.1) was used to identify the Wnt domain and DIX domain containing proteins separately. All candidates were checked manually in the SMART database^2^ and the InterPro database^3^ separately, and these redundant sequences were removed (Letunic and Bork, 2018; Mitchell et al., 2019). Furthermore, we used the same methods to search the Wnt proteins and DIX proteins in 23 other species, including 11 vertebrates: *Homo sapiens*, *Bos taurus*, *Capra hircus*, *Ornithorhynchus anatinus*, *Gallus gallus*, *Anser cygnoides*, *Struthio camelus*, *Gekko japonicus*, *Python bivittatus*, *Xenopus laevis* and *Danio rerio*; 12 invertebrates: *Octopus bimaculoides*, *Strongylocentrotus purpuratus*. *Drosophila hydei*, *Musca domestica*, *Aedes aegypti*, *Trichoplusia ni*, *Bombyx mori*, *Leptinotarsa decemlineata*, *Apis mellifera*, *Onthophagus taurus*, *Caenorhabditis elegans*, and *Amphimedon queenslandica*. The molecular weights (Mw) and isoelectric points (pI) of these identified Wnt and DIX proteins were calculated in the ExPASy server. The significant differences between the Mw and pI of Wnt and DIX proteins were checked separately through one-way ANOVA with Dunnett’s multiple comparisons method, and a *p* value smaller than 0.05 was considered significant. Pearson’s correlation was calculated based on the numbers of Wnt and DIX genes in different species.

### Chromosomal Distributions and Phylogenetic Analyses of Wnt and DIX Family Members

The chromosomal distributions of all mouse Wnt and DIX genes were obtained from the NCBI genome database and MGI database. Then these genes were marked in corresponding chromosomes according to their physical positions in respective chromosomes, and the coordinate diagram was built by MapChart software (Voorrips, 2002). The Wnt and DIX genes were labeled in orange and blue respectively. Those genes closely connected on chromosomes and have no spacer genes were taken as tandem duplication genes. For phylogenetic analyses, the sequences of Wnt and DIX proteins from different species were aligned through the ClustalW method separately, and then the neighbor-joining (N-J) phylogenetic trees of Wnt and DIX proteins were built with 1000 replicate bootstraps in MEGA software (version X).

### Ortholog Analyses

Orthologous analyses of mouse Wnt and DIX genes were performed as previously reported (Zou et al., 2016). Orthologs of mouse genes in yeast (*Saccharomyces cerevisiae*), nematodes (*Caenorhabditis elegans*), sea urchins (*Strongylocentrotus purpuratus*) and zebrafish (*Danio rerio*) were identified from the InParanoid8 online server^4^. According to the status of ortholog existence, the genes were classified into different phylogenetic patterns. Specifically, the status of orthologs in “zebrafish, sea urchin, nematode and yeast,” “only in zebrafish, sea urchin and nematode,” “only in zebrafish and sea urchin,” “only in zebrafish,” and “none of the other four species,” are represented by the symbols of “++++,” “+++ −,” “++ −−,” “+ −−−,” and “−−−−” respectively. The different phylogenetic patterns can be used to indicate the different times of evolutionary stages.

### Domain Organization

Domain organization was performed as previously described (Zhao et al., 2020b). The conserved domains of mouse Wnt and DIX proteins were identified in the Pfam server with the default parameters, the identified domains were furtherly examined in NCBI CDD database^5^. Then the domain distributions of the mouse LIM protein were visualized in the Evolview server (Subramanian et al., 2019).

### Molecular Co-evolution Analyses

The Mirrortree server^6^ was used to assess the co-evolution between Wnt and DIX protein families (Ochoa and Pazos, 2010; Ochoa et al., 2015). The complete protein sequences of 6 DIX proteins and 19 Wnt proteins were retrieved from the Uniprot database^7^ and a total of 114 Wnt-DIX sequence pairs were used to compute coevolution correlation coefficients separately.

### Gene Expression Patterns Analyses

To analyze the expression patterns of mouse Wnt genes and DIX genes in the heart tissue during cardiac hypertrophy, we downloaded the gene expression data (accessions: GSE30428 and GSE56348) from the NCBI GEO database (Kreymborg et al., 2010; Lai et al., 2014). The title of GSE30428 is called “Identification of right heart-enriched genes in a murine model of chronic outflow tract obstruction,” which contains the gene expression data of the right ventricles of six TAC operated mice and two sham operated mice. Right ventricle hypertrophy was induced through the PAC method. The gene expression levels were measured by using the Affymetrix Mouse Gene 1.0 ST Array (MoGene-1_0-st) and the output data were processed and analyzed through affy (version 1.62.0) and limma (version 3.41.0) packages in R software (version 4.0). Furthermore, we analyzed the gene expression data at 1, 3 and 6 weeks after PAC operation. On the other hand, the title of GSE56348 is called “Gene expression microarray profiling in mice hearts with pathological and physiological cardiac hypertrophy.” It contains the gene expression profiles of the left ventricle of mice with transverse aortic constriction (TAC) induced hypertrophy (chronic hypertrophy, CH), vs sham-operated control (Sham-CH). Both CH and Sham-CH group contain five female C57Bl6/J mice, and the gene expression levels were measured using the Affymetrix Mouse Gene 1.0 ST Array (MoGene-1_0-st). The expression data was analyzed 4 weeks after TAC or Sham surgical operations. The Affymetrix probe level data was processed using Robust Multi-Array (RMA) to get normalized scores of expression level for each probe. After the expression profiles of Wnt and DIX genes were obtained, the pheatmap package of R software (version: 4.0) was employed to draw the heatmaps.

### Primary and Tertiary Structural Analyses of Wnt and DIX Proteins

We downloaded the tertiary structures of human Wnt-3 (PDB id: 6ahy), Xenopus Wnt-8 (4f0a), Drosophila Wnt-D (4krr), rat Axin_DIX (1wsp), zebrafish Dixin_DIX (5y3c), mouse Dvl1_DIX (3pz8) and human Dixin_DIX (4wip) from the PDB website. The 4krr is a partial structure of drosophila Wnt, and then we built a complete structural model based on this partial structure through the I-TASSER server (Yang et al., 2015). Then, structural alignments were carried out as previously described (Gai et al., 2016). Amino acid sequence alignment of the Wnt and DIX domains in different species was carried out using the ClustalW method. Identical residues and residues with similar properties were marked in red and yellow, respectively. The root-mean-square deviations (RMSD) between each structure were calculated in VMD software. Furthermore, the evolutionary conservation of residues in these tertiary structures was estimated in the Consurf server^8^. All 3D structural figures were generated by PyMOL (version 1.74).

## Data Availability Statement

The datasets presented in this study can be found in online repositories. The names of the repository/repositories and accession number(s) can be found in the article/Supplementary Material.

## Author Contributions

ZG, JZ, and GG designed the study and wrote the manuscript. ZG, YW, and LT preformed bioinformatic analyses. YW and LT preformed statistical analyses. All authors read and approved this manuscript.

## Conflict of Interest

The authors declare that the research was conducted in the absence of any commercial or financial relationships that could be construed as a potential conflict of interest.

## Footnotes

1 http://pfam.xfam.org

2 http://smart.embl-heidelberg.de

3 http://www.ebi.ac.uk/interpro/

4 http://inparanoid.sbc.su.se/cgi-bin/index.cgi

5 https://ncbiinsights.ncbi.nlm.nih.gov/tag/conserved-domains-database-cdd/

6 http://csbg.cnb.csic.es/mtserver/help.html

7 https://www.uniprot.org

8 https://consurf.tau.ac.il
