# Supplementary material for: Whole Genome Level Analysis of the Wnt and DIX Gene Families in Mice and Their Coordination Relationship in Regulating Cardiac Hypertrophy
Source: Front Genet. 2021 Jun 8;12:608936. doi: 10.3389/fgene.2021.608936 (PMC8217762; doi:10.3389/fgene.2021.608936)
Supplement: Supplementary file 1 [file Data_Sheet_1.PDF]

Supplementary Files

Whole Genome Level Analysis of the Wnt and DIX Gene Families in Mice and Their Coordination Relationship in Regulating Cardiac Hypertrophy

Zhongchao Gai, Yujiao Wang, Jieqiong Zhao

Contents

Table S1. Characteristics of Wnt genes in mouse

Table S2. Characteristics of DIX genes in mouse

Table S3. List of Wnt and DIX genes in different phylogenetic patterns

Table S4. List of the co-evolution correlation between Wnt and DIX proteins

Figure S1. The significant difference test of Mw and pI between these Wnt and DIX proteins from different species

Table S1. Characteristics of Wnt genes in mouse

Gene name	Chromosome	start	end	mRNA	aa
Wnt8b	19	44493472	44514273	1107	368
Wnt8a	18	34542327	34548061	1065	354
Wnt5b	6	119432531	119544347	1080	359
Wnt6	1	74771892	74785322	1095	364
Wnt2b	3	104945272	104961921	1170	389
Wnt16	6	22288227	22298522	1095	364
Wnt9a	11	59306928	59333552	1098	365
Wnt10a	1	74792019	74804176	1254	417
Wnt2	6	17988940	18030585	1083	360
Wnt3	11	103774150	103817957	1068	355
Wnt9b	11	103727364	103749821	1080	359
Wnt10b	15	98770712	98778150	1170	389
Wnt7b	15	85535437	85581821	1062	353
Wnt11	7	98835084	98854747	1065	354
Wnt1	15	98789857	98793830	1113	370
Wnt4	4	137277635	137299501	1056	351
Wnt7a	6	91363981	91411353	1050	349
Wnt3a	11	59248036	59290751	1059	352
Wnt5a	14	28504750	28527448	1143	380

Table S2. Characteristics of DIX genes in mouse

	Chromosome	Start	End	mRNA	aa
Dixdc1	9	50662752	50739517	2136	711
Dvl3	16	20516982	20534010	2151	716
Dvl2	11	70000592	70010109	2211	736
Axin2	11	108920349	108950783	2523	840
Dvl1	4	155847402	155859303	2088	695
Axin1	17	26138688	26195811	2499	832

Table S3. List of Wnt and DIX genes in different phylogenetic patterns

	"++++"	"+++-"	"++--"	"+---"	"----"
Wnt genes		Wnt-4	Wnt-1	Wnt-11	Wnt-2
		Wnt-3	Wnt-10a		
		Wnt-7b	Wnt-6		
		Wnt-3a	Wnt-9a		
		Wnt-5a	Wnt-8b		
		Wnt-2b	Wnt-9b		
		Wnt-5b	Wnt-8a		
		Wnt-16			
		Wnt-10b			
		Wnt-7A			
DIX genes		Axin1			
		Axin2			
		DVL1			
		DVL2			
		DVL3			
		Dixin			

Table S4 List of the co-evolution correlation between Wnt and DIX proteins

	**Dixdc1**	**Dvl1**	**Dvl2**	**Dvl3**	**Axin1**	**Axin2**
Wnt-1	0.998	0.836	0.832	0.629	0.9	0.301
Wnt-2	0.999	0.836	0.803	0.669	0.919	0.375
Wnt-2b	0.846	0.829	0.835	0.691	0.874	0.221
Wnt-3	0.975	0.655	0.605	0.635	-0.077	0.844
Wnt-3a	0.235	0.69	0.601	0.617	-0.16	0.78
Wnt-4	0.762	0.684	0.582	0.611	-0.008	0.861
Wnt-5a	0.993	0.823	0.813	0.672	0.94	0.311
Wnt-5b	0.959	0.722	0.751	0.565	0.797	0.755
Wnt-6	0.645	0.685	0.63	0.755	0.136	0.862
Wnt-7a	0.141	0.637	0.548	0.573	-0.144	0.768
Wnt-7b	0.997	0.644	0.578	0.578	-0.054	0.858
Wnt-8a	0.778	0.908	0.858	0.828	0.929	0.231
Wnt-8b	0.925	0.827	0.793	0.721	-0.041	0.858
Wnt-9a	0.954	0.723	0.554	0.684	0.008	0.888
Wnt-9b	0.934	0.661	0.541	0.641	0.145	0.931
Wnt-10a	0.823	0.93	0.782	0.896	0.831	0.563
Wnt-10b	0.77	0.647	0.568	0.727	0.256	0.882
Wnt-11	-0.264	0.527	0.526	0.381	0.255	-0.091
Wnt-16	0.993	0.849	0.807	0.688	0.967	0.29
**Average**	0.761210526	0.742789474	0.684578947	0.661105263	0.393315789	0.604631579

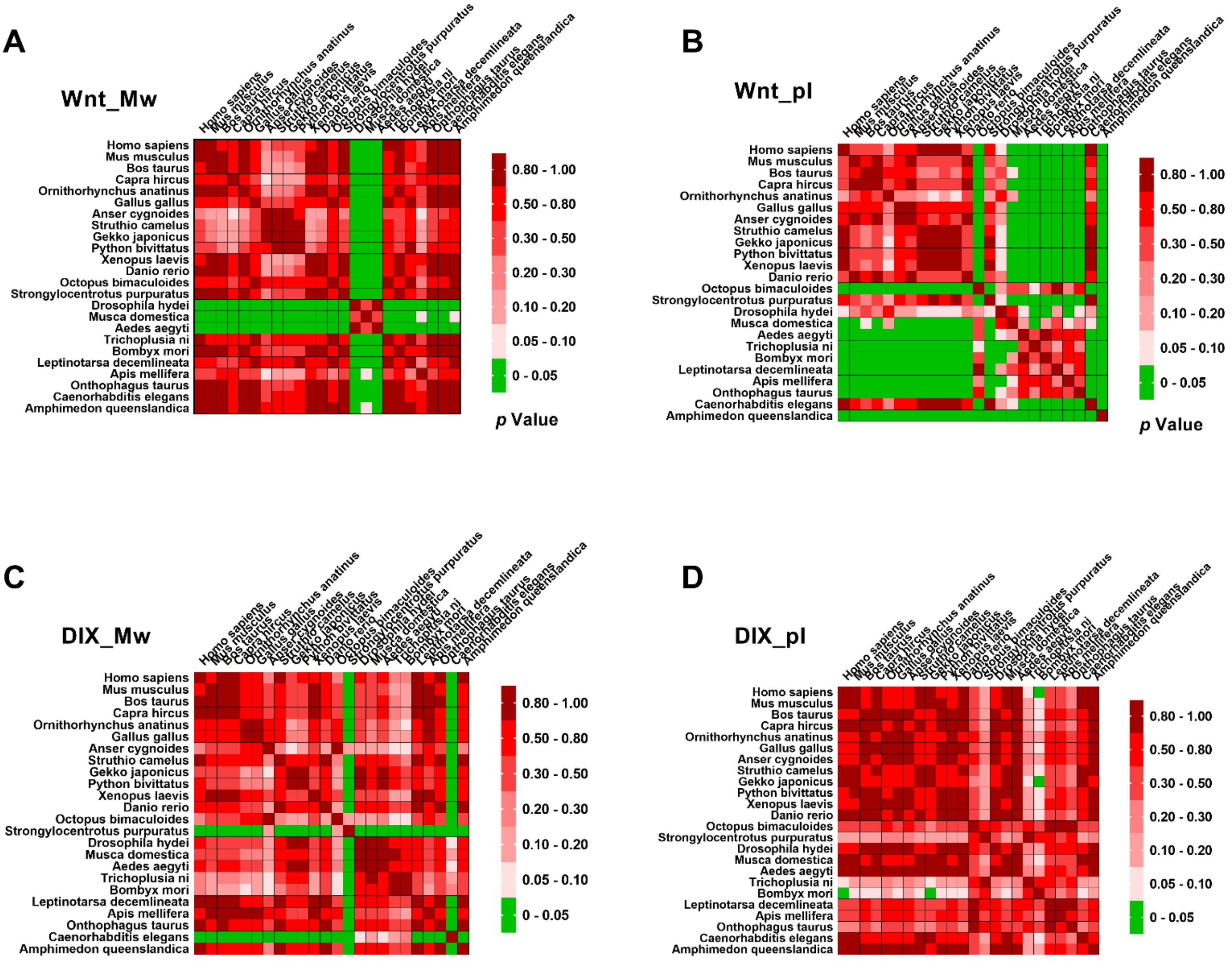

Figure S1. The significant difference test of Mw and pI between these Wnt and DIX proteins from different species. Molecular weight of Wnt proteins (A) and DIX protein (B) from different species. Isoelectric point (pI) distributions of Wnt proteins (C) and DIX proteins (D) from different species. Scale bar indicates the students' t test values.
